# Cyanobacterial Neurotoxin β-*N*-Methylamino-L-alanine (BMAA) in Shark Fins

**DOI:** 10.3390/md10020509

**Published:** 2012-02-21

**Authors:** Kiyo Mondo, Neil Hammerschlag, Margaret Basile, John Pablo, Sandra A. Banack, Deborah C. Mash

**Affiliations:** 1 Department of Neurology, Miller School of Medicine, University of Miami, Miami, FL 33136, USA; Email: kmondo@med.miami.edu (K.M.); mbasile@med.miami.edu (M.B.); jpp71@hotmail.com (J.P.); 2 Rosensteil School of Marine and Atmospheric Science and Policy, University of Miami, Miami, FL 33149, USA; Email: nhammerschlag@rsmas.miami.edu; 3 Leonard and Jayne Abess Center for Ecosystem Science and Policy, University of Miami, Coral Gables, FL 33124, USA; 4 RJ Dunlap Marine Conservation Program, University of Miami, Miami, FL 33149, USA; 5 Institute for Ethnomedicine, Box 3464, Jackson Hole, WY 83001, USA; Email: sandra@ethnomedicine.org

**Keywords:** β-*N*-methylamino-L-alanine, neurotoxin, neurodegenerative disease, cyanobacteria, elasmobranch, conservation

## Abstract

Sharks are among the most threatened groups of marine species. Populations are declining globally to support the growing demand for shark fin soup. Sharks are known to bioaccumulate toxins that may pose health risks to consumers of shark products. The feeding habits of sharks are varied, including fish, mammals, crustaceans and plankton. The cyanobacterial neurotoxin β-*N*-methylamino-L-alanine (BMAA) has been detected in species of free-living marine cyanobacteria and may bioaccumulate in the marine food web. In this study, we sampled fin clips from seven different species of sharks in South Florida to survey the occurrence of BMAA using HPLC-FD and Triple Quadrupole LC/MS/MS methods. BMAA was detected in the fins of all species examined with concentrations ranging from 144 to 1836 ng/mg wet weight. Since BMAA has been linked to neurodegenerative diseases, these results may have important relevance to human health. We suggest that consumption of shark fins may increase the risk for human exposure to the cyanobacterial neurotoxin BMAA.

## 1. Introduction

Sharks are apex predators in virtually all marine environments and impact ecosystem structure and function through trophic cascades [1,2]. However, shark populations are experiencing global declines as a result of over-fishing, largely driven to support the burgeoning shark fin trade [3,4,5]. A minimum of 26 to 73 million sharks per year, representing a combined weight of 1.7 million tons are killed in both target and bycatch fisheries to support the high demand for fins in Asian markets [6]. High exploitation rates continue to increase annually driven by the rising demand for highly prized fins used to make shark fin soup, an Asian delicacy and one of the world’s most expensive fishery products [7]. Shark fins consist of cartilage with fibrous protein collagens that add texture and consistency to the soup. The larger the fin and higher fin needle content (collagen fibers), the more expensive the soup. Sharks accumulate mercury and other heavy metals [8] that pose health risks to consumers of shark products, including shark fin soup.

The neurotoxin BMAA is produced by diverse species of free-living cyanobacteria found in terrestrial and aquatic environments [9] and cyanobacterial symbionts [10]. BMAA has been linked to the development of neurodegenerative brain diseases, such as Alzheimer’s disease and Amyotrophic Lateral Sclerosis (ALS) [11,12]. Cyanobacteria are found in lakes, rivers, estuaries, and marine waters with bloom growth increased due to nutrient loading from agricultural and industrial runoff, farm animal wastes, sewage, groundwater inflow and atmospheric deposition [13]. The occurrence of BMAA has been reported in isolated cyanobacteria from waters in the Baltic Sea [14], China [15], Holland [16], South Africa [17], British Island [18], and Peru [19] as well as in laboratory cultures of free-living marine cyanobacteria [20].

BMAA has been measured in high concentration in marine fish and invertebrates collected from South Florida coastal waters [21] and the Baltic Sea [14]. Given the ubiquity of cyanobacteria in marine ecosystems, BMAA could bioaccumulate up the marine food web to sharks, potentially posing health risks to consumers of shark products.

Given the increasing exploitation of sharks and the potential health hazard associated with bioaccumulation of BMAA in marine food webs, we conducted a study to determine if BMAA could be detected in shark fins. Specifically, we sampled fins and select organs from seven common shark species found in South Florida waters (USA) for analysis and detection of BMAA using multiple analytical techniques.

## 2. Results and Discussion

The fins of seven shark species collected in South Florida coastal waters (Table 1) were analyzed by high performance liquid chromatography with fluorescence detection (HPLC-FD). BMAA was detected in a total acid hydrolysate using HPLC-FD and validated by triple quadrupole liquid chromatography tandem mass spectrometry (LC/MS/MS). Precolumn derivatization of the amino acids in the sample was performed using the fluorescent tag 6-aminoquinolyl-*N*-hydroxysuccinimidyl carbamate (AQC). AQC universally tags amino acids at primary and secondary nitrogens producing complex molecules that do not degrade during high pressure separation [22].

**Table 1:** Shark specimens and location sites with presence and absence of cyanobacteria blooms indicated.

Species	Scientific Name	Location		Month	Cyanobacterial Blooms
Blacknose ^a^	*Carcharhinus acronotus*	25.62099°N	80.15602°W	August	not present
Blacktip ^b^	*Carcharhinus limbatus*	25.00644°N	80.99969°W	March	present
Blacktip ^b^	*Carcharhinus limbatus*	25.00644°N	80.99969°W	September	present
Blacktip ^a^	*Carcharhinus limbatus*	25.59968°N	80.15205°W	July	not present
Blacktip ^b^	*Carcharhinus limbatus*	25.01109°N	80.99832°W	September	present
Blacktip ^b^	*Carcharhinus limbatus*	25.00644°N	80.99969°W	March	present
Blacktip ^a^	*Carcharhinus limbatus*	25.62592°N	80.15442°W	October	not present
Blacktip ^a^	*Carcharhinus limbatus*	25.61905°N	80.1714°W	October	not present
Blacktip ^a^	*Carcharhinus limbatus*	25.64757°N	80.1881°W	April	not present
Blacktip ^a^	*Carcharhinus limbatus*	25.67199°N	80.18144°W	September	not present
Blacktip ^b^	*Carcharhinus limbatus*	25.01089°N	81.00419°W	September	present
Blacktip ^b^	*Carcharhinus limbatus*	25.00976°N	81.00079°W	September	present
Blacktip ^b^	*Carcharhinus limbatus*	25.01715°N	81.01056°W	September	present
Bonnethead ^a^	*Sphyrna tiburo*	25.36711°N	80.14806°W	March	not present
Bonnethead ^a^	*Sphyrna tiburo*	25.36711°N	80.14806°W	March	not present
Bonnethead ^a^	*Sphyrna tiburo*	25.40807°N	80.21806°W	October	not present
Bull ^b^	*Carcharhinus leucas*	25.01715°N	81.01056°W	September	present
Bull ^b^	*Carcharhinus leucas*	25.01309°N	81.00129°W	September	present
Great Hammerhead ^a^	*Sphyrna mokarran*	25.62138°N	80.15656°W	July	not present
Great Hammerhead ^b^	*Sphyrna mokarran*	25.01715°N	81.01056°W	September	present
Lemon ^b^	*Negaprion brevirostris*	25.00644°N	80.99969°W	June	present
Lemon ^b^	*Negaprion brevirostris*	25.00644°N	80.99969°W	June	present
Nurse ^a^	*Ginglymostoma cirratum*	25.61942°N	80.1835°W	September	not present
Nurse ^b^	*Ginglymostoma cirratum*	24.88335°N	80.84475°W	April	present
Nurse ^b^	*Ginglymostoma cirratum*	25.00644°N	80.99969°W	March	present
Nurse ^a^	*Ginglymostoma cirratum*	25.62311°N	80.15626°W	August	not present
Nurse ^a^	*Ginglymostoma cirratum*	25.60062°N	80.15214°W	August	not present
Nurse ^a^	*Ginglymostoma cirratum*	25.60569°N	80.1534°W	August	not present
Nurse ^a^	*Ginglymostoma cirratum*	25.62311°N	80.15626°W	August	not present

^a^ Biscayne Bay; ^b^ Florida Bay.

**Figure 1:**
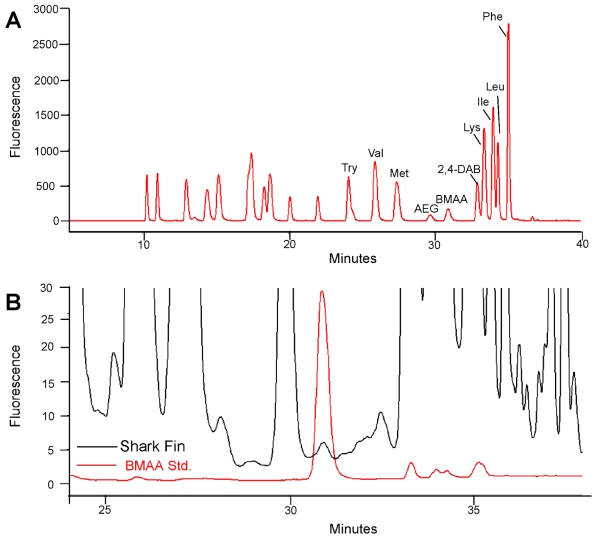
HPLC identification of BMAA in shark fins. (**A**) HPLC-FD separation of non-hydrolyzed AQC derivatized amino and diamino acids: tyrosine (Try), valine (Val), methionine (Met), *N*-2(amino)ethylglycine (AEG), β-*N*-methylamino-L-alanine (BMAA), and 2,4-diaminosuccinic acid (2,4-DAB), lysine (Lys), isoleucine (Ile), leucine (Leu), phenylalanine (Phe); (**B**) Representative chromatogram of great hammerhead shark fin (black) overlaid with BMAA standard (red). Separation of the derivatized amino and diamino acids was optimized on a C18 column.

The AQC-derivatized BMAA standard elutes closest to methionine (Met). Figure 1A illustrates the HPLC-FD separation of the standard amino acids, BMAA and its isomers *N*-2(amino)ethylglycine (AEG) and 2,4-diaminosuccinic acid (2,4-DAB). The relative retention time for BMAA (30.89 min) was clearly separated from AEG (29.67 min) and 2,4-DAB (32.91 min).

**Figure 2:**
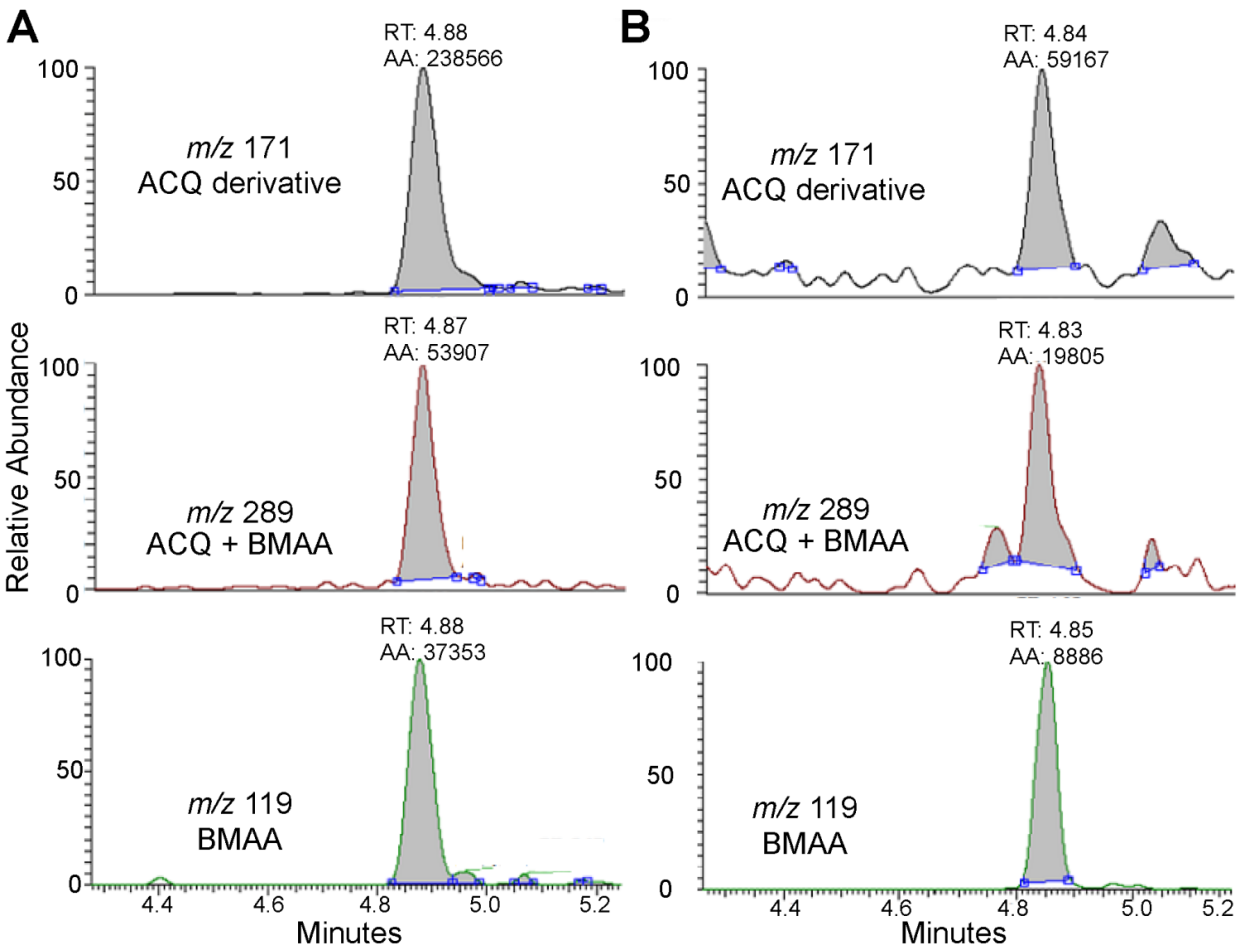
LC/MS/MS identification and verification of BMAA in a single great hammerhead shark fin from South Florida Bay waters. (**A**) Triple quadrupole LC/MS/MS verification of BMAA standard. The chromatographic spectra of the three major ions produced from collision-induced dissociations of *m/z* 459 are: (top panel) protonated AQC derivative fragment (*m/z* 171), the quantitation ion; (center panel) protonated-BMAA AQC fragment (*m/z* 289), the first qualifier ion and (lower panel) protonated-BMAA fragment (*m/z* 119), the second qualifier ion; (**B**) Representative triple quadrupole LC/MS/MS verification of BMAA in a great hammerhead shark. Spectra are the same as in Column A.

These results demonstrate that BMAA did not coelute with any of the natural or diamino acids contained in the shark matrix. A representative HPLC-FD chromatogram of a great hammerhead shark fin sample shown in Figure 1B illustrates the BMAA peak. BMAA in the shark sample shown in Figure 1 was confirmed using triple quadrupole LC/MS/MS (Figure 2). The mass spectrometric verification of the BMAA peak confirms HPLC detection of BMAA in the shark sample [9,16,17,23]. The product ions with masses of *m/z* 171, 289, and 119 were detected in the third quadrupole for both the sample and the BMAA standard and the ratio of the three fragmentation product ions were within normal variation as described previously [18].

We detected and quantified BMAA in the fins of all shark species with concentrations ranging from 144 to 1836 ng/mg wet weight (Table 2). BMAA was not detected in six out of the total number (*n* = 29) of individual fin clip specimens assayed. The results demonstrate high concentrations of BMAA in shark fins collected in areas with or without active cyanobacteria blooms. We observed considerable variability within the same shark species having a similar body length and taken from the same collection sites. For example, the bonnethead shark had BMAA concentrations that ranged from 320 to 1836 ng/mg over a range of only 76 to 79 cm. Of the 7 members of the elasmobranch family surveyed, both the nurse shark and the blacktip shark had fin clip samples where BMAA was not detected (Table 2). Interestingly, the two samples taken from nurse sharks sampled in Florida Bay were positive for BMAA while only one of the five sampled from Biscayne Bay had a quantifiable peak (Table 2). There was no apparent correlation of BMAA concentration with the size of the shark or lifespan at sampling.

**Table 2:** BMAA concentrations in shark fins from South Florida coastal waters.

Species	Size (cm)	BMAA Mean (ng/mg)	SE	BMAA (ng/100 cm shark)
Blacknose ^a^ (1)	120	1,663		1,386
Blacktip ^b,^* (4)	61	280	84	460
Blacktip ^b,^* (4)	99	144	18	210
Blacktip ^a^ (1)	162	ND		ND
Blacktip ^b,^* (1)	165	ND		ND
Blacktip ^b,^* (1)	173	286		165
Blacktip ^a^ (1)	174	168		97
Blacktip ^a^ (1)	177	247		140
Blacktip ^a^ (1)	148	794		537
Blacktip ^a^ (1)	155	811		522
Blacktip ^b,^* (1)	165	303		184
Blacktip ^b,^* (1)	165	745		453
Blacktip ^b,^* (1)	168	252		150
Bonnethead ^a^ (4)	76	632	96	860
Bonnethead ^a^ (4)	79	320	59	408
Bonnethead ^a^ (4)	77	1,836	364	2,385
Bull ^b,^* (4)	163	232	60	142
Bull ^b,^* (4)	183	264	96	144
Great Hammerhead ^a^ (4)	247	1,528	212	619
Great Hammerhead ^b,^* (4)	175	528	211	291
Lemon ^b,^* (4)	168	556	210	332
Lemon ^b,^* (4)	201	628	66	312
Nurse ^a^ (1)	226	223		99
Nurse ^b,^* (1)	213	169		79
Nurse ^b,^* (1)	168	161		96
Nurse ^a^ (1)	165	ND		ND
Nurse ^a^ (1)	235	ND		ND
Nurse ^a^ (1)	207	ND		ND
Nurse ^a^ (1)	241	ND		ND

Number in parentheses indicates sample size; SE: standard error; ND: not detected; ^a^ Biscayne Bay; ^b^ Florida Bay; * Active cyanobacterial blooms.

We measured BMAA using HPLC-FD in the organs and muscles of great hammerhead sharks killed as a result of recreational fishing activities. As shown in Table 3, BMAA was detected in kidney, liver, and muscle but was not measured in the heart tissue for this species. The highest levels were observed in the kidney, suggesting that uptake and excretion of BMAA along with other natural amino acids occurs in this organ. Although the heart sample had no detectable BMAA, further studies are needed to rule out possible accumulation of BMAA in contractile cardiac tissue.

**Table 3:** BMAA concentrations in different tissues of great hammerhead sharks (*Sphyrna mokarran*) collected in South Florida coastal waters.

Organ	BMAA Mean (ng/mg)	SE	BMAA (ng/100 cm of shark)
Kidney (3)	1450	687	598
Liver (4)	588	81	243
Fin (8)	1028	211	487
Muscle (3)	58	41	24
Heart (2)	ND		ND

Number in parentheses indicates sample size, SE: standard error, ND: not detected.

Cyanobacterial blooms in South Florida coastal waters occurred in the 1980s and have persisted ever since [21]. Most cyanobacteria are known to produce the neurotoxin BMAA that has been linked to development of the neurodegenerative brain diseases [10,11,24]. Brand *et al*. [21] recently reported that BMAA was detected in several species of crustaceans and fish from the same South Florida coastal waters surveyed in the present study. These marine species are part of the diet of some groups of sharks. Since sharks are at the highest trophic level, they may bioaccumulate BMAA from active exposure to cyanobacterial bloom sites. All seven shark species analyzed in this study had BMAA detected in high amounts in their fins. Interestingly, high concentrations of BMAA were detected in the fins of some sharks collected in areas that had no active cyanobacteria blooms. Sharks are highly migratory, making it likely that they pass in and out of areas where cyanoblooms may have occurred over time [21,25]. While planktonic cyanobacteria are abundant, benthic and cyanobacteria epiphytic on seagrass and macroalgal blades are also present, providing a source of BMAA from the lowest trophic levels to higher animals within the same marine ecosystem.

The bonnethead shark that had the highest levels of BMAA in this study are known to primarily feed on members of the benthic zone, including blue crabs and pink shrimps which reportedly have very high concentrations of BMAA (mean concentration of 2505 µg/g and 2080 µg/g, respectively [21]). Sharks as long-lived apex predators may concentrate protein-associated BMAA over time in certain tissues. This pattern of bioaccumulation is what has been observed for mercury and other heavy metal toxins in sharks across the lifespan [8]. The range of BMAA concentrations measured in the different sharks surveyed most likely reflect their ecological niches, different foraging patterns, and their size and age differences.

BMAA was measured in select organ tissues including the kidney, liver, and muscle of the great hammerhead shark (*Sphyrna mokarran*). The tissue uptake of BMAA has been previously reported in the brain and muscle of bottom-dwelling fishes in the Baltic Sea [14], muscle and tissues from fish and crustaceans in South Florida coastal waters [21], and in brain, muscle, skin, intestine, kidney and fur in flying foxes from Guam [23]. Taken together, these studies suggest that BMAA may be misincorporated into proteins where it bioaccumulates with repeat exposures.

Shark fins consist of cartilage with fibrous protein collagens. Shark fin cartilage powder or capsules are marketed as dietary supplements and claimed to combat and/or prevent a variety of illnesses. However, the benefits of this supplement have not been significantly proven, nor has shark cartilage been reviewed by the US Food and Drug Administration (FDA). Recently Field *et al*. [26] hypothesized that collagen abnormality in the skin of sporadic ALS patients may be caused by the misincorporation of BMAA leading to misfolding of the collagen proteins. In keeping with this hypothesis, the highest levels of BMAA found in the Guam flying fox were detected in skin tissue known to contain collagen as a major component [23].

The elevated level of BMAA in shark fins provides additional support that marine cyanobacteria may represent a route for human exposure to BMAA. Further studies are needed to confirm this finding and to demonstrate that widespread BMAA detections in sharks may occur outside of South Florida coastal waters. The recent finding that BMAA co-occurs with other cyanotoxins in contaminated water supplies raises the possibility that low-level human exposure to BMAA exists in many parts of the world [17]. The possible link between BMAA and gene/environment interactions in progressive neurodegenerative diseases [9] warrants concern for exposure to BMAA in human diets. In Asia, shark fin soup is considered a delicacy, which drives a high consumer demand for this product. Our report suggests that human consumption of shark fins may pose a health risk for BMAA exposure especially if it occurs with mercury or other toxins.

## 3. Experimental Section

### 3.1. Sample Collection

Archived shark fins were collected in South Florida (USA) from various areas with or without documented cyanobacterial blooms as described previously [21]. Fin clips were sampled during coastal shark surveys in Florida Bay and Biscayne Bay (Table 1). Sharks were temporarily caught using circle-hook drumlines (a modified fishing apparatus). Drumline units are composed of a base weight that is anchored to the sea floor, outfitted with 75 feet of 700 pound test monofilament, attached by a swivel to a 4-strand 900 pound test circle hook gangion, which permits captured sharks to swim in large circles around the stationary base weight. Sharks were brought alongside the vessel for non-lethal tissue collection, whereby a 2 × 2 cm clip was removed from the trailing edge of the first dorsal fin and a 4 mm muscle biopsy sampled from the hepaxial muscle on the shark’s left flank, after which the animal was released. Specimens were immediately frozen and archived. An opportunistic sample of fin, muscle, liver, heart, and kidney were obtained from dead animals killed as a result of recreational fishing activities. Tissue specimens from nurse (*Ginglymostoma cirratum*), blacktip (*Carcharhinus limbatus*), great hammerhead (*Sphyrna mokarran*), bull (*Carcharhinus leucas*), blacknose (*Carcharhinus acronotus*), lemon (*Negaprion brevirostris*) and bonnethead (*Sphyrna tiburo*) sharks were included in this survey (Table 1).

### 3.2. Fluorescence HPLC Methods for Analysis of Protein-Associated BMAA

BMAA was detected and quantified using a previously validated HPLC method with minor modifications [20,27]. Shark fin clips and tissues were hydrolyzed for 18 h in 6 N HCl (1:8 wt/v) at 110 °C. Hydrolysates were filtered at 15,800 × g for 3 min and concentrated in a speed-vac (Thermo-Savant SC250DDA Speed Vac Plus with a Savant refrigerator trap RVT 4104). The dried extract was resuspended in 0.1 M trichloroacetic acid then washed with chloroform for removal of any residual lipids. The washed extract and standards were derivatized with 6-aninoquinolyl-*N*-hydroxysuccinimidyl carbamate (AQC) using the AccQ-Fluor reagent (Waters Crop, Millford, MA) and BMAA was separated from the protein amino acids by reverse-phase high pressure chromatography (Waters Nova-Pak C18 column, 3.9 mm × 300 mm) eluted in a gradient of 140 mM sodium acetate, 5.6 mM triethylamine, pH 5.2 (mobile phase A), and 52% (v/v) acetonitrile in water (mobile phase B) at 37 °C using a flow rate of 1.0 mL/min, and 10 µL sample injection volume. The samples were eluted using a 60 min gradient: 0.0 min = 100% A; 2 min = 90% A curve 11; 5 min = 86% A curve 11; 10 min = 86% A curve 6; 18 min = 73% A curve 6; 30 min = 57% A curve 10; 35 min = 40% A curve 6; 37.5 min = 100% B curve 6; 47.5 min = 100% B curve 6; 50 min = 100% A curve 6; 60 min = 100% A curve 6. Detection of the AQC fluorescent tag was achieved using a Waters 2475 Multi λ-Fluorescence Detector with excitation at 250 nm and emission at 395 nm. Experimental shark samples were compared with standard spiked shark fin matrix negative for endogenous BMAA containing a commercial BMAA reference standard (Sigma B-107; >95% purity, St. Louis, MO, USA). The limits of detection (LOD) and limits of quantification (LOQ) were 2.7 and 7.0 ng, respectively. The percentage of recovery of BMAA was 88%.

### 3.3. Triple Quadrupole LC/MS/MS

Identification of a BMAA peak detected by reverse-phase HPLC was verified by liquid chromatography/mass spectrometry/mass spectrometry (LC/MS/MS) using product ion mode in a triple quadrupole system. The frozen shark fin tissues were hydrolyzed for 18 h in 6 N HCl at 110 °C and then dried in a Thermo-Savant SC250DDA Speed Vac Plus (Waltham, MA, USA). The sample was reconstituted in dilute HCl (20 mM) and derivatized with AQC, which increased the molecular weight of the BMAA analyte from 118 to 458. The derivatized sample was separated using gradient elution at 0.65 mL/min in aqueous 0.1% (v/v) formic acid (Eluent A) and 0.1% (v/v) formic acid in acetonitrile (Eluent B): 0.0 min = 99.1% A; 0.5 min = 99.1% A curve 6; 2 min = 95% A curve 6; 3 min = 95% A curve 6; 5.5 min = 90% curve 8; 6 min = 15% A curve 6; 6.5 min = 15% A curve 6; 6.6 min = 99.1% A curve 6; 8 min = 99.1% A curve 6. Nitrogen gas was supplied to the heated electrospray ionization (H-ESI) probe with a nebulization pressure of 40 psi and a vaporizer temperature of 400 °C. The mass spectrometer was operated under the following conditions: the capillary temperature was set at 270 °C, capillary offset of 35, tube lens offset of 110, auxiliary gas pressure of 35, spray voltage 3500, source collision energy of 0, and multiplier voltage of −1719. A divert valve was used during the clean-up and equilibration parts of the gradient. The second quadrupole was pressurized to 1.0 Torr with 100% argon. Product-ion analysis of BMAA used *m/z* 459 as the precursor ion for collision induced dissociation (CID) and thereby all other ions were excluded in the first quadrupole. Further two-step mass filtering was performed during selective reaction monitoring (SRM) of BMAA after CID in the second quadrupole, monitoring the following transitions: *m/z* 459 to 119, CE 21 eV; *m/z* 459 to 289 CE 17 eV; *m/z* 459 to 171 CE 38 eV. The resultant three product ions originating from derivatized BMAA (*m/z* 119, 289, 171) were detected after passing the third quadrupole and their relative abundances were quantified.

## 4. Conclusions

BMAA can be transferred from cyanobacteria in the lower trophic levels (teleosts and crustaceans) to marine apex predators. Sharks are among the most threatened marine vertebrates [28] due in part to the high demand of their fins for dietary and medicinal purposes. The consumption of shark products that contain the cyanotoxin BMAA could increase risk for development of neurodegenerative diseases, including Alzheimer’s disease and ALS [11,24]. The worldwide prevalence of Alzheimer’s disease is estimated to quadruple in 2050 by which time 1 in 85 persons worldwide will be living with the disease [29]. Until more is known about the possible link of BMAA to Alzheimer’s disease and other neurodegenerative diseases, it may be prudent to limit exposure of BMAA in the human diet. Our report suggests that consumption of shark fins increases the risk for human exposure to BMAA, a neurotoxic amino acid that accumulates in biological tissues.
